# Pneumococcal Hemolytic Uremic Syndrome in Children in Sweden

**DOI:** 10.1001/jamanetworkopen.2025.5721

**Published:** 2025-04-17

**Authors:** Helena Hildenwall, Valya Georgieva, Joachim Luthander, Susanne Westphal Ladfors, Lisa Sartz, Milan Chromek

**Affiliations:** 1 Division of Pediatrics, Department of Clinical Science, Intervention and Technology, Karolinska Institutet, Stockholm, Sweden; 2 Astrid Lindgren Children’s Hospital, Karolinska University Hospital, Stockholm, Sweden; 3 Centre for Health and Sustainability, Department of Women’s and Children’s Health, Uppsala University, Uppsala, Sweden; 4 Department of Global Public Health, Karolinska Institutet, Stockholm, Sweden; 5 Department of Women’s and Children’s Health, Karolinska Institutet, Sweden; 6 Department of Pediatrics, Institute of Clinical Science, Sahlgrenska Academy, University of Gothenburg, Gothenburg, Sweden; 7 Queen Silvia Children’s Hospital, Gothenburg, Sweden; 8 Section for Pediatric Nephrology, Skåne University Hospital, Department of Clinical Sciences, Lund University, Lund, Sweden

## Abstract

This cohort study of children in Sweden reviewed pneumococcal hemolytic uremic syndrome cases between 2009 and 2024 for incidence rates and serotype data.

## Introduction

Hemolytic uremic syndrome (HUS) is a rare but severe condition presenting with nonimmune microangiopathic hemolytic anemia, thrombocytopenia, and acute kidney injury. Most cases occur in young children, peaking between 6 months and 4 years of age. The most common cause for HUS is infection with Shiga toxin–producing *Escherichia coli*, but other Shiga toxin–producing bacteria including *Shigella spp* and *Citrobacter spp* have also been isolated.^1^ Cases may also occur with *Streptococcus pneumoniae* but are estimated to represent less than 5% of all HUS cases.^2^ The introduction of pneumococcal conjugate vaccines (PCV) has contributed to decrease the incidence of pneumococcus HUS (pHUS), especially after introduction of the 13-valent vaccine (PCV13), which covers serotype 19A, the most common serotype in pHUS.^3^ In Sweden, PCV13 was introduced in the National Immunization Program (NIP) in 2010 but replaced by 10-valent vaccine (PCV10) in 2019. The annual number of pHUS cases has been between zero to 2 since 2009 among Sweden’s approximately 550 000 children below 5 years of age. In 2023, the number of cases increased to 8, prompting further assessments.

## Method

In this cohort study, we reviewed all pHUS cases among Swedish children from January 2009 to December 2024 (25 cases in total) focusing on immunization coverage and pneumococcal serotype. Patients were identified through the 3 pediatric nephrology centers treating pHUS: Astrid Lindgren Children’s Hospital in Stockholm, Queen Silvia Children’s Hospital in Gothenburg, and Skåne University Hospital in Lund. Clinical data were retrieved from patient files with pHUS cases defined according to clinical and laboratory findings.^4^ Serotype data were provided from the Swedish Public Health Authority. Ethics approval was obtained from the Central Ethical Research Board, Sweden. This included a waiver of individual consent as the research involved deidentified retrospective data analysis with no direct patient interaction. The report follows the Strengthening the Reporting of Observational Studies in Epidemiology (STROBE) reporting guideline for cohort studies. Data were analyzed using STATA version 16 (StataCorp LLC)

## Results

Of the 25 children included (median [IQR] age, 19 months [16-22 months]; 13 female [52%]), 17 (68%) occurred between 2022 and 2024 (Table). The incidence among children aged 0 to 5 years was 0.11 cases per 100 000 children per year from 2009 to 2021, compared with 1.09 cases per 100 000 children per year in 2022 to 2024. The median (IQR) age at diagnosis was 19 months (16-22 months). One death occurred in a child with an underlying severe chronic condition. Three children were unimmunized, and 17 children had received PCV10. Blood culture results were available for 23 of 25 children, with *S pneumoniae* growth found in 15 of those 23 children. Urine pneumococcal antigen was analyzed from 17 cases and detected in all. Direct antiglobulin tests were positive in 15 children. Of 15 available serotypes, 10 were identified as serotype 3, and 5 were identified as serotype 19A, with the annual distribution presented in the Figure.

**Table.** Characteristics of Children With Pneumococcal Hemolytic Uremic Syndrome in Sweden, 2009-2024

Characteristics	Children, No. (%)			
	All (N = 25)	Diagnosed 2009-2021 (n = 8)		Diagnosed 2022-2024 (n = 17)
Age at diagnosis, median (IQR), mo	19 (16-22)	18 (16-26)	19 (17-22)	
Sex				
Female	13 (52)	5 (63)	8 (47)	
Male	12 (48)	3 (38)	9 (53)	
Pneumococcal vaccine type				
PCV10	17 (68)	3 (38)	14 (82)	
PCV13/PCV15	2 (8)	1 (13)	1 (6)	
Unvaccinated	3 (12)	2 (25)	1 (6)	
Unknown	3 (12)	2 (25)	1 (6)	
Serotype				
Not identified	10 (40)	4 (50)	6 (35)	
3	10 (40)	3 (38)	7 (41)	
19A	5 (20)	1 (13)	4 (24)	

Abbreviation: PCV, pneumococcal conjugate vaccine valence.

**Figure.**
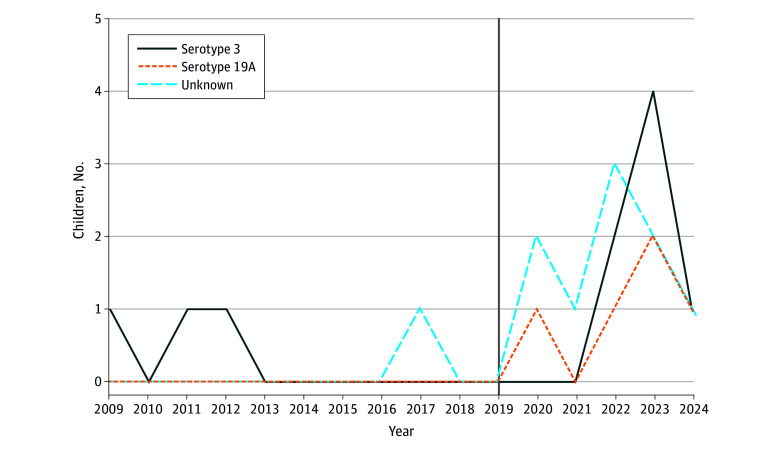
Pneumococcal Hemolytic Uremic Syndrome Serotype Distribution The vertical line indicates change from pneumococcal conjugate vaccine valence 13 (PCV13) to PCV10 in the Swedish National Immunization Program.

## Discussion

We report an increase in pHUS incidence in Swedish children with serotype 3 as the main pathogen. While serotype 3 may cause illness despite immunization, the rise occurred after the switch in the NIP from PCV13, covering serotype 3, to PCV10, in which serotype 3 and 19A are uncovered. In comparison, a national surveillance in England showed a decrease in pHUS incidence after replacing PCV7 with PCV13.^3^

The rise may also relate to post–COVID-19 epidemiological changes, with a reported increase of invasive pneumococcal disease (IPD) following a global decline during the pandemic.^5^ However, it is unclear if COVID-19 affected the serotype distribution of IPD, as no serotype shift has been observed.^6^ While serotype 3 has been previously reported as a main pathogen for IPD, 19A has been the dominant serotype identified in pHUS.^4^

Study limitations include a retrospective small sample with some missing data that restrain hypothesis testing. We conclude that the pHUS incidence has recently increased in Sweden, with serotype 3 identified in most cases. A recent switch to PCV15 in the NIP in 2024 may halt the observed increase, but given the risks for serotype shift, continued surveillance of pHUS and IPD remains essential.

## Notes

Supplement.
Data Sharing Statement
