# Supplementary material for: Pneumococcal Hemolytic Uremic Syndrome in Children in Sweden
Source: JAMA Netw Open. 2025 Apr 17;8(4):e255721. doi: 10.1001/jamanetworkopen.2025.5721 (PMC12006866; doi:10.1001/jamanetworkopen.2025.5721)
Supplement: Supplement. — Data Sharing Statement [file jamanetwopen-e255721-s001.pdf]

Data Sharing Statement

Hildenwall. Pneumococcal Hemolytic Uremic Syndrome in Children in Sweden. *JAMA Netw Open*. Published April 17, 2025. doi:10.1001/jamanetworkopen.2025.5721

Data
Data available: Yes
Data types: Deidentified participant data
How to access data: Data can be requested from the corresponding author helena.hildenwall@ki.se
When available: With publication

Supporting Documents
Document types: None

Additional Information
Who can access the data: Anyone requesting the data
Types of analyses: any purpose
Mechanisms of data availability: after approval of a proposal
